# Genetic diversity of the E Protein of Dengue Type 3 Virus

**DOI:** 10.1186/1743-422X-6-113

**Published:** 2009-07-23

**Authors:** Alberto A Amarilla, Flavia T de Almeida, Daniel M Jorge, Helda L Alfonso, Luiza A de Castro-Jorge, Nadia A Nogueira, Luiz T Figueiredo, Victor H Aquino

**Affiliations:** 1 Virology Research Center, School of Medicine of Ribeirão Preto/USP, Ribeirão Preto – SP, Brazil; 2 Department of Clinical, Toxicological and Bromatological Analysis, FCFRP/USP, Ribeirão Preto – SP, Brazil; 3 Bioinformatics Laboratory, Department of Genetics, School of Medicine of Ribeirão Preto/USP, Ribeirão Preto – SP, Brazil; 4 Department of Toxicological and Clinical Analysis, Federal University of Ceara, Brazil

## Abstract

**Background:**

Dengue is the most important arbovirus disease in tropical and subtropical countries. The viral envelope (E) protein is responsible for cell receptor binding and is the main target of neutralizing antibodies. The aim of this study was to analyze the diversity of the E protein gene of DENV-3. E protein gene sequences of 20 new viruses isolated in Ribeirao Preto, Brazil, and 427 sequences retrieved from GenBank were aligned for diversity and phylogenetic analysis.

**Results:**

Comparison of the E protein gene sequences revealed the presence of 47 variable sites distributed in the protein; most of those amino acids changes are located on the viral surface. The phylogenetic analysis showed the distribution of DENV-3 in four genotypes. Genotypes I, II and III revealed internal groups that we have called lineages and sub-lineages. All amino acids that characterize a group (genotype, lineage, or sub-lineage) are located in the 47 variable sites of the E protein.

**Conclusion:**

Our results provide information about the most frequent amino acid changes and diversity of the E protein of DENV-3.

## Background

During the first decades of the 20^th ^century, dengue was considered a sporadic disease, causing epidemics at long intervals. However, dramatic changes in this pattern have occurred and, currently, dengue is the most important mosquito-borne viral disease worldwide. Approximately, 3 billion people are at risk of acquiring dengue viral infections in more than 100 countries in tropical and subtropical regions. Annually, it is estimated that 100 million cases of DF and half a million cases of dengue DHF/DSS occur worldwide resulting in approximately 25,000 deaths [1]. Dengue disease can be caused by any of the four antigenically related viruses named dengue virus type 1, 2, 3 and 4 (DENV-1, -2, -3 and -4). All of these serotypes can cause a large spectrum of clinical presentations, ranging from asymptomatic infection to dengue fever (DF) and to the most severe form, dengue haemorrhagic fever/dengue shock syndrome (DHF/DSS). Early diagnosis of dengue virus infection, which can be achieved by detecting a viral protein or genome, is important for patient management and control of dengue outbreaks [2].

Dengue is an enveloped virus with a single-stranded, positive-sense RNA genome of about 11 kb containing a single open reading frame, flanked by untranslated regions (5' and 3' UTR) [3]. The viral RNA encodes a single polyprotein, which is co- and pos-translationally cleaved into 3 structural (C, prM and E) and 7 nonstructural proteins (NS1-NS2A-NS2B-NS3-NS4A-NS4B-NS5) proteins [4]. The envelope (E) glycoprotein is the major component of the virion external surface, responsible for important phenotypic and immunogenic properties. E protein is a multifunctional protein, which is involved in cell receptor binding and virus entry via fusion with host cell membranes. Thus, E protein is the main target of neutralizing antibodies [5-10]. The crystal structure analysis of this protein revealed that it includes three domains (I, II, and III) that exhibit significant structural conservation when compared to other flaviviruses [11]. For flaviviruses, most of amino acid residues related to host range determinant, tropism and virulence are located in domain III [12,13].

Similar to other RNA viruses, DENV exhibit a high degree of genetic variation due to the non-proofreading activity of the viral RNA polymerase, rapid rates of replication, immense population size, and immunological pressure [14]. Historically, variants within each DENV serotype have been classified in different ways, accompanying technological progress. Studies from the seventies showed the existence of antigenic variants within DENV-3 showing that DENV-3 strains from Puerto Rico and Tahiti were antigenically and biologically different from those of Asia [15]. In the eighties, the term "topotype", based on RNA fingerprinting, was used to define five genetic variants within DENV-2 [16,17]. Other molecular methods such as cDNA-RNA hybridization, hybridization using synthetic oligonucleotides, and restriction endonuclease analysis of RT-PCR products were also used to demonstrate the existence of genetic variability within each serotype [18-22]. In the nineties, the use of nucleic acid sequencing methods and phylogenetic analysis allowed the identification of different genomic groups, called "genotypes" or "subtypes", within each DENV serotype [23-25]. Today, several geographically distinct genotypes are described within each serotype. Thus, DENV-1 includes five genotypes: genotype I contains viruses from the Americas, Africa, and Southeast Asia; genotype II includes a single isolate from Sri Lanka; genotype III includes a strain from Japan isolated in 1943; genotype IV includes strains from Southeast Asia, the South Pacific, Australia, and Mexico; and genotype V group contains viruses from Taiwan and Thailand [23,26,27]. DENV-2 encompasses six genotypes denominated Asian I, Asian II, American, American/Asian, Cosmopolitan and Sylvatic [23,24,28]. DENV-3 was classified into four genotypes: genotype I comprises viruses from Indonesia, Malaysia, Philippines and the South Pacific islands; genotype II comprises viruses from Thailand; genotype III is represented by viruses from Sri Lanka, India, Africa and America; genotype IV comprises Puerto Rican viruses. Recently, it has been suggested that exist an additional group that was named genotype V [25,29]. DENV-4 was classified into two genetically distinct genotypes. Genotype I includes viruses from the Philippines, Thailand and Sri Lanka; genotype II includes viruses from Indonesia, Tahiti, Caribbean Islands (Puerto Rico, Dominica) and Central and South America [30]. A third genotype of DENV-4 was identified which includes sylvatic isolates that formed a distinct genotype [27].

Increased numbers of DENV sequences in the GenBank has given a better picture of the genetic diversity of these viruses, suggesting the existence of intragenotipic groups within each genotype. Identification of these groups will lead to a better understanding of the migration pattern of the viruses, as well as the detection of emergent viruses with altered antigenicity, virulence, or tissue tropism. In this study, we have analyzed the variability of the E protein gene of DENV-3 by comparison of new and GenBank deposited sequences and found several lineage and sub-lineages within the different genotypes.

## Results

Nucleotide sequences of the E protein gene (1479 bp) of 20 DENV-3 strains isolated in Ribeirao Preto and 427 sequences retrieved from the GenBank were included in this study. These sequences represent viruses isolated between 1956 and 2007. After an initial analysis, 75 identical sequences, three recombinant strains, two mutants, one rare, and five sequences corresponding to the same five strains deposited with different access codes were excluded from the study (Additional file 1) [29,31]. Thus, 361 sequences were used to analyze the E protein diversity and the phylogenetic relationship of the viruses.

To analyze the diversity of the E protein, nucleotide sequences were aligned and compared. Any of the 1479 sites in the alignment were considered a variable site when at least one virus showed a nucleotide substitution at that site. By this criteria, 634 variable sites were found to be evenly distributed in the E protein gene; 157 of these showed non-synonymous substitutions (substitutions in the codon that induce amino acid changes) (Additional file 2). Seventy non-synonymous substitutions sites were observed only in one virus, 28 sites in two viruses and 59 sites in three or more viruses.

**Figure 1:**
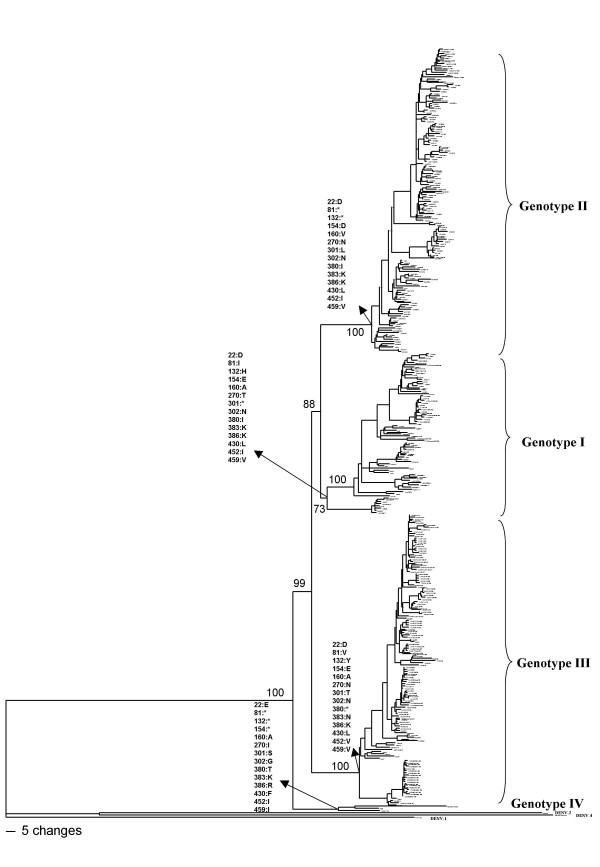
**DENV-3 phylogenetic tree based on the E gene sequences**. The three was constructed using the method of Neighbor-joining with 1000 bootstrap replications. The genotypes are labeled according to the scheme of Lanciotti (1994) and the amino acid changes distinguishing each genotype are shown on the tree. Protein E gene sequences of DENV-1, DENV-2 and DENV-4 were used as outgroup. Branch lengths are proportional to percentage of divergence. Tamura Nei (TrN+I+G) nucleotide substitution model was used with a proportion of invariable sites (I) of 0.3305 and gamma distribution (G) of 0.9911. Bootstrap support values are shown for key nodes only.

Based on the aligned nucleotide sequences, several phylogenetic analysis including maximum parsimony and distance methods were performed and all approaches yielded identical or nearly identical topologies. The phylogenetic tree showed four genetic groups within the DENV-3 (Figure 1) where genotype I was represented by strains from Indonesia, Malaysia, Philippines and the South Pacific islands; genotype II included mainly isolates from Thailand; genotype III was represented mainly by viruses from Sri Lanka and Latin America and genotype IV comprised Puerto Rican viruses.

For a better characterization of the genetic groups, E protein gene sequences of all viruses were compared manually. As mentioned above, 634 variable sites were observed within the 1479 nucleotides of the E protein gene (Additional file 2). Variable sites with nucleotide substitutions in at least 90% of the members of any genotype were considered informative sites. Thus, 95 of the 634 were considered informative sites. Among these 95, 18 sites were in the domain I of E protein, 28 in domain II, 27 in domain III, and 22 in the transmembrane domain (Additional file 3). Each genotype showed a characteristic nucleotide sequence when the informative sites were analyzed. Nucleotide substitution in the informative sites was mostly due to transitions (80 sites, 81%) rather than transversions (21 sites, 19%). Nucleotide substitution were more frequent in the 3rd position (74 sites, 78%) of the codon, followed by the first position (15 sites, 16%) and finally, the second position (6 sites, 6%). Non-synonymous substitutions were observed in 14 (15%) of the 95 informative sites (residues 22, 81, 132, 154, 160, 270, 301, 302, 380, 383, 386, 430, 452 and 459). Three non-synonymous substitutions were identified in domain I, three in domain II, five in domain III, and three in the transmembrane domain (Additional file 3). Based on the tertiary structure of the E protein of DENV-3 (36), it was observed that amino acid residues 81, 132, 154, 270, 301, 302, 380, and 383 were located in solvent-exposed loops. Residues 22 and 386 were located in β-strands exposed on the viral surface. The residue 160 was located in a hydrophobic region. Residues 430, 452 and 459 were located in the transmembrane region (Additional file 4A).

### Intragenotipic groups

Careful analysis of the topology of the phylogenetic tree suggests the existence of intragenotipic groups (Figure 1). To better characterize these internal groups, protein E gene sequences of members of each genotype were independently analyzed.

### Genotype I

**Figure 2:**
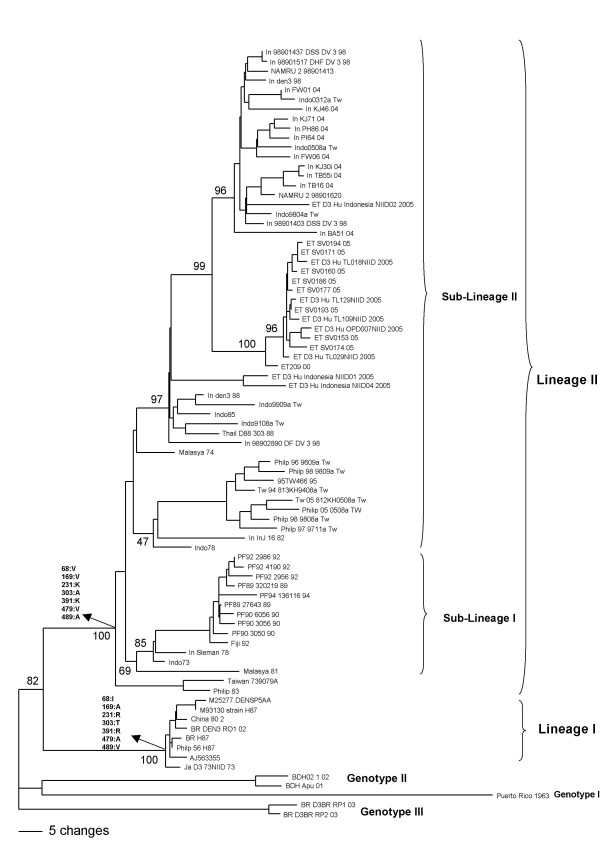
**Genotype I phylogenetic tree constructed using the method of Neighbor-joining with 1000 bootstrap replications**. Sequences of each genotype II, III and IV were used as outgroup. Branch lengths are proportional to percentage divergence. Tamura Nei (TrN+I+G) nucleotide substitution model was used with a proportion of invariable sites (I) of 0.5420 and gamma distribution (G) of 2.6122. The lineage and sub-lineages are marked. Amino acids changes are indicated on the tree. Bootstrap support values are shown for key nodes only.

A phylogenetic tree was constructed using 76 protein E gene sequences of genotype I viruses (Figure 2). The tree showed that these viruses form two different clades that were denominated lineage I and II. The nucleotide sequence comparison showed the presence of 348 variable sites in the 1479 nucleotides of the E protein gene with 40 of them considered informative sites. Non-synonymous substitutions were observed in seven informative sites (Table 1). Amino acid residues 231, 303 and 391 were found to be located in solvent-exposed loops, residues 68 and 169 in hydrophobic regions (Additional file 4B). Residues 479 and 489 were located in the transmembrane region.

**Table 1:** Nucleotide and amino acid substitutions in the informative sites of genotype I.

**Nucleotide**						**Protein**				**Domains**
	
		**Genotype I**								
							
**Position**		**Lineage**		**Lineage II**		**Position**	**Lineagen**		**Type of amino acid Changes**	
				**Sub-Lineage**						
			
**Gene**	**Codon**	**I**	**II**	**I**	**II**	**Protein**	**I**	**II**		I
		
48	3	G	A							
135	3	T	C							
	
174	3	G	A							II
202	1	A	G			68	I	V	Conservative	
219	3	A	G							
222	3	T	C							
282	3	T	C							
342	3	G	A							
366	3	A	G							
393	3	A	G							
	
441	3			T	C					I
474	3	T	C							
506	2	C	T			169	A	V	Conservative	
516	3	T	C							
537	3	C	T							
	
588	3	A	G							II
633	3	C	T							
640	1	T	C							
645	3	C	T							
663	3			A	G					
684	3	T	C							
692	2	G	A			231	R	K	Conservative	
714	3	T	C							
735	3			G	A					
759	3	A	G							
777	3	T	C							
	
849	3			T	C					I
	
909	1	A	G			303	T	A	Nonconservative	III
912	3			C	T					
1101	3	T	A							
1153	1	C	T							
1172	2	G	A			391	R	K	Conservative	
	
1269	3	G	A							TM
1281	3	G	A							
1302	3	C	G							
1317	3	G	A							
1329	3			A	G					
1380	3	C	T							
1436	2	C	T			479	A	V	Conservative	
1466	2	T	C			489	V	A	Conservative	

Domain I: 1–156^nt ^(1–52^aa^); 397–573^nt ^(133–191^aa^); 835–882^nt ^(279–294^aa^)

Domain II: 157–396^nt ^(53–132^aa^); 574–834^nt ^(192–278^aa^)

Domain III: 883–1176^nt ^(295–392^aa^)

Domain TM: 1177–1479^nt ^(393–493^aa^)

nt:are indicated the nucleotide positions

aa::are indicated the amino acid positions

The phylogenetic tree showed that lineage II included two sub-lineages (Figure 2). The comparison of nucleotide sequences (n = 68) showed the presence of 318 variable sites within members of this lineage, six of them being informative sites with synonymous substitutions (Table 1).

### Genotype II

**Figure 3:**
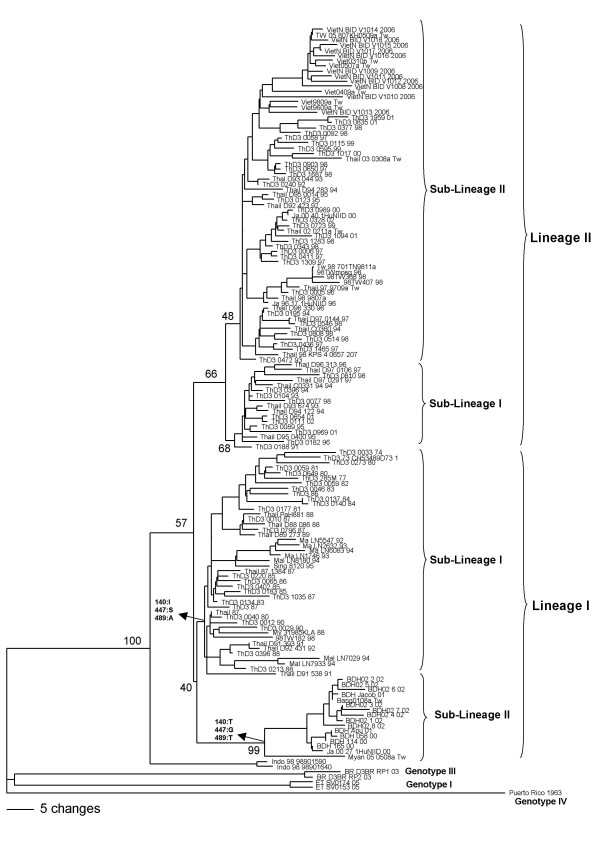
**Genotype II phylogenetic tree constructed using the method of Neighbor-joining with 1000 bootstrap replications**. Sequences of each genotype I, III and IV were used as outgroup. Branch lengths are proportional to percentage divergence. Tamura Nei (TrN+I+G) nucleotide substitution model was used with a proportion of invariable sites (I) of 0.5041 and gamma distribution (G) of 1.3902. The lineage and sub-lineages are marked. Amino acids changes are indicated on the tree. Bootstrap support values are shown for key nodes only.

Genotype II included 144 viruses that were grouped into two lineages (Figure 3). Comparison of these sequences showed 392 variable sites; four of them being informative sites with synonymous substitutions (Table 2). Lineage I included 62 sequences that form two sub-lineages with 255 variable sites; 17 of them were considered informative sites and three had non-synonymous substitutions (Table 3). The amino acid residue 140 was located in a β-strand exposed in the surface of the protein; residues 447 and 489 were in the transmembrane domain (Additional file 4C). Lineage II included 83 viruses distributed in two sub-lineages. The comparison of these sequences showed 275 variable sites with only two informative sites, which showed synonymous substitutions (Table 2).

**Table 2:** Nucleotide and amino acid substitutions in the informative sites of genotype II.

**Nucleotide**								**Protein**				**Domains**
		
		**Genotype II**										
							
				**Lineage I**		**Lineage II**		**Position**	**Lineage I**			
									
**Position**		**Lineage**		**Sub-Lineage**		**Sub-Lineage**			**Sub-Lineage**		**Type of amino acid Changes**	
			
**Gene**	**Codon**	**I**	**II**	**I**	**II**	**I**	**II**	**Protein**	**I**	**II**		
	
54	3			T	A							I
90	3					C	T					
96	3					T	C					
	
273	3			A	G							II
351	3			G	A							
	
419	2			T	C			140	I	T	Nonconservative	I
549	3			C	T							
525	3	A	G									
558	3			G	C							
	
609	3	A	C									II
708	3	G	A									
747	3			T	C							
834	3			T	C							
	
963	3			G	A							III
1002	3	T	C									
1134	3			G	C							
1176	3			T	A							
	
1188	3			C	C							TM
1233	3			A	T							
1339	1			T	G			447	S	G	Nonconservative	
1436	2			G	C							
1465	1			A	A							
1467	3			T	T			489	A	T	Nonconservative	

Domain I: 1–156^nt ^(1–52^aa^); 397–573^nt ^(133–191^aa^); 835–882^nt ^(279–294^aa^)

Domain II: 157–396^nt ^(53–132^aa^); 574–834^nt ^(192–278^aa^)

Domain III: 883–1176^nt ^(295–392^aa^)

Domain TM: 1177–1479^nt ^(393–493^aa^)

nt:are indicated the nucleotide positions

aa::are indicated the amino acid positions

**Table 3:** Nucleotide and amino acid substitutions in the informative sites of genotype III.

**Nucleotide**								**Domains**
	
		**Genotype III**						
			
				**Lineage I**		**Lineage II**		
					
**Position**		**Lineage**		**Sub-Lineage**		**Sub-Lineage**		
	
**Gene**	**Codon**	**I**	**II**	**I**	**II**	**I**	**II**	
	
96	3					C	T	I
117	3					C	A	
121	1	C	T					
157	1					C	T	
312	3	T	A					
423	3	T	C					
	
588	3	A	G					II
633	3	C	T					
672	3					C	T	
784	1	C	T					
825	3					C	T	
	
1050	3	C	T					II
1131	3					A	G	
1170	3					C	T	
	
1185	3			G	T			TM
1314	3	T	C					
1356	3	G	A					
1374	3	T	A					
1473	3	A	G					

Domain I: 1–156^nt ^(1–52^aa^); 397–573^nt ^(133–191^aa^); 835–882^nt ^(279–294^aa^)

Domain II: 157–396^nt ^(53–132^aa^); 574–834^nt ^(192–278^aa^)

Domain III: 883–1176^nt ^(295–392^aa^)

Domain TM: 1177–1479^nt ^(393–493^aa^)

nt:are indicated the nucleotide positions

aa::are indicated the amino acid positions

### Genotype III

**Figure 4:**
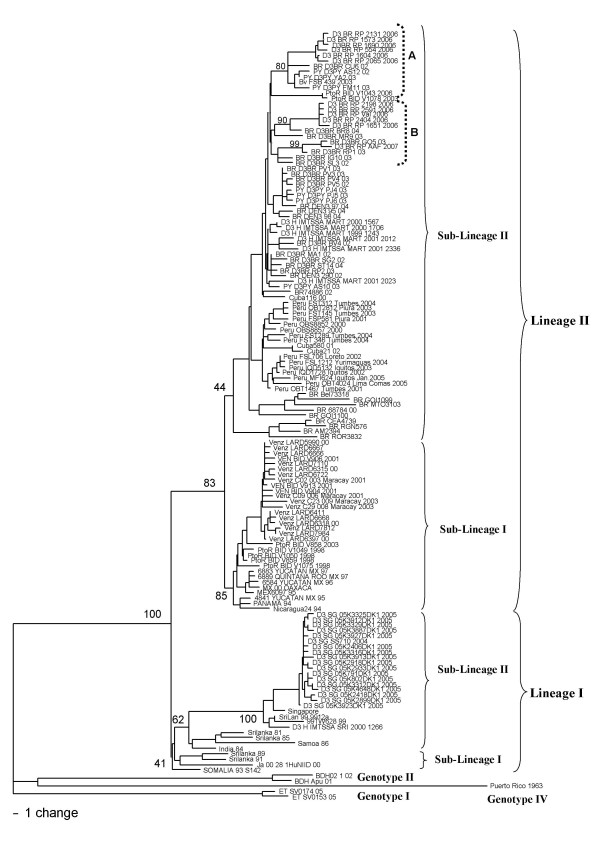
**Genotype III phylogenetic tree constructed using the method of Neighbor-joining with 1000 bootstrap replications**. Some viruses of each genotype I, II and IV were used as outgroup. Branch lengths are proportional to percentage divergence. Tamura Nei (TrN+G) nucleotide substitution model was used with gamma distribution (G) of 0.2796. The Lineage and Sub-lineages are marked. Amino acids changes are indicated on the tree. Bootstrap support values are shown for key nodes only.

Genotype III was composed of 138 sequences grouped in two lineages (Figure 4). Sequences comparison showed 321 variable sites with 11 informative sites, all of them with synonymous substitutions. Lineage I included 29 sequences grouped into sub-lineage I and II with 123 variable sites with only one of them considered as informative site, which showed a synonymous substitution (Table 3). The lineage II included 108 sequences forming two groups, sub-lineage I and II; these sequences showed 250 variable sites and only seven of them were considered as informative sites, all of them were synonymous substitutions (Table 3). The sub-lineage II of lineage II included the 20 viruses isolated in Ribeirao Preto, SP, Brazil, between 2006–2007. These viruses were more closely related to those isolated in other regions of Brazil than to viruses that circulated in Ribeirao Preto, in 2003 (D3BR/RP1/2003 and D3BR/RP2/2003). They formed two groups, one more closely related to the strain D3BR/CU6/2002 isolated in Cuiabá close to the border with Bolivia (Group A) and another more closely related to the strain D3BR/BR8/2004 isolated in northern Brazil (Group B). Only the strain D3BR/RPAAF/2007 isolated in 2007 was more closely related to D3BR/RP1/2003 strain.

## Discussion

The comparison of E protein gene sequences of DENV-3 revealed many variable sites; however, only 47 of them showed nucleotide substitutions that induced amino acid changes in a significant number of viruses (Additional file 5). Therefore, the E protein of DENV-3 showed 47 sites with variable amino acid residues, which were located mainly on the viral surface. Our molecular modeling analysis showed that all the amino acid substitutions do not interfere with the conformational structure of the E protein. These polymorphic amino acid residues could be involved in cell attachment, viral pathogenesis, and recognition by neutralizing antibodies [12,13,32]. Recently, it was shown that a panel of sera collected from DF and DHF patients 16–18 month after illness had different levels of neutralizing antibodies to different DENV-3 strains [33]. Those authors used in the neutralization tests isolates from Cuba and Puerto Rico, which showed amino acid substitutions at several of the 47 variable sites (Additional file 6). This suggests that those residues may be involved in neutralization differences, but further studies are necessary to confirm this hypothesis.

The phylogenetic analysis, based on E protein gene sequences, presented in this study showed that DENV-3 are distributed into four genotypes which is supported by complete mapping of this gene, and is in agreement with previous studies [25,34]. In addition, internal groups (lineages and sub-lineages) were observed within genotypes I, II and III. It was not possible to confirm internal sub-grouping within the genotype IV due to the low number of sequences available in the GenBank. All amino acids that characterize a group (genotype, lineage, or sub-lineage) are located in the 47 variable sites of the E protein. Characteristic amino acid residues corresponding to the different DENV-3 genotypes, lineages, and sub-lineages are evenly distributed in the E protein, and most of them are exposed on the viral surface.

Recently, it has been reported the existence of a group of virus forming another genotype (genotype V) within DENV-3 [29]. However, our phylogenetic and nucleotide/amino acid substitution analysis suggest that those viruses of genotype V form a sub-group within the clade of genotype I and for this reason we have name this subgroup as lineage I. The phylogenetic trees generated in other studies using maximum likelihood and bayesian methods showed that the so-called genotype V is in the same clade of genotype I [35,36]. Therefore, we propose the maintenance of the classification of DENV-3 into four genotypes as previously suggested [25,34].

Other authors have also observed the existence of some of the intragenotypic groups described in this study. It has been observed that genotype I includes three groups of viruses: South Pacific, Philippines, and East Timor viruses [37]. South Pacific viruses are included in the sub-lineage I, while Philippines and East Timor are internal groups within our sub-lineage II of genotype I. It has also been suggested that genotype II includes two groups of viruses called: pre- and post-1992 [29]. These groups correspond to our lineages I and II of genotype II, respectively. The post-1992 viruses include groups A and B, which correspond to our sub-lineages I and II of lineage II. In addition, it has been suggested that isolates from Bangladesh form a distinct group within genotype II [38]. This group corresponds to our sub-lineage II of lineage I. Another study has also found three internal groups within genotype II: Malaysia, Bangladesh and Vietnam viruses [37]. These groups correspond to our sub-lineage I of lineage I, sub-lineage II of lineage I, and sub-lineage II of lineage II, respectively. The genotype III viruses have been classified into four groups: Latin America, East Africa and groups A and B from Sri Lanka viruses [39]. Our analysis showed a similar distribution of genotype III viruses; however, we found that Latin America viruses (lineage II) form two groups that we called sub-lineages I and II. These sub-lineages showed also internal monophyletic groups, which were omitted to simplify the classification. However, other authors have identified these internal groups within sub-lineages I and II [37,40-42].

All the DENV-3 isolated in Ribeirao Preto between 2006–2007 were grouped within the sub-lineage II/lineage II of genotype III. They were more closely related to viruses isolated in other cities than to those that were previously reported at Ribeirao Preto in 2003, suggesting that DENV-3 is constantly moving within the country [43]. Brazil is a large tropical country with optimal conditions for the spread of dengue virus making difficult the control of the disease.

In summary, our results provide information about the most frequent amino acid changes in the E protein of DENV-3. These amino acids could be involved in cell attachment, virus pathogenesis, and recognition by neutralizing antibodies. However, further studies are needed to confirm these hypotheses. The phylogenetic relationship suggested the existence of only four genotypes of DENV-3. In addition, we observed internal groups within genotypes I, II and III.

## Methods

### Virus and RNA purification

Twenty DENV-3 strains isolated in C6/36 cells (passage number 2) from DF and DHF/DSS patients, between 2006–2007, in Ribeirao Preto city, Brazil, were included in this study. Viral RNA was purified from 140 μl of culture fluid with the QIAamp Viral RNA kit (Qiagen, Germany), following manufacturer's recommendations.

### RT-PCR and sequencing

The E protein gene of the samples were reverse-transcribed and amplified by polymerase chain reaction (RT-PCR), using consensus primers, as previously described [43]. The amplicons were purified from agarose gel using the QIAquick Gel Extraction Kit (Qiagen, USA), and directly sequenced in an ABI PRISM^®^3100 Genetic Analyzer (Applied Biosystems, USA). The sequences obtained in this study were submitted to the GenBank and registered with the following accession numbers: D3_BR/RP/1573/2006 (EU617019), D3_BR/RP/1604/2006 (EU617020), D3_BR/RP/1625/2006 (EU617021), D3_BR/RP/1651/2006 (EU617022), D3_BR/RP/2065/2006 (EU617023), D3_BR/RP/2131/2006 (EU617024), D3_BR/RP/2170/2006 (EU617025), D3_BR/RP/2198/2006 (EU617026), D3_BR/RP/2404/2006 (EU617027), D3_BR/RP/2591/2006 (EU617028), D3_BR/RP/2604/2006 (EU617029), D3_BR/RP/554/2006 (EU617030), D3_BR/RP/590/2006 (EU617031), D3_BR/RP/597/2006 (EU617032), D3_BR/RP/AAF/2007 (EU617033), D3_BR/RP/Val/2006 (EU617034), D3BR/RP/549/2006 (EU617035), D3BR/RP/1690/2006 (EU617036), D3BR/RP/2121/2006 (EU617037), D3BR/RP/2167/2006 (EU617038).

### Phylogenetic analysis of sequences

The E protein gene sequences (1479 bp) obtained in this study were analyzed using the Vector NTI software (Informatix, USA) and then aligned with 427 sequences of DENV-3 retrieved from GenBank (Additional file 1) using the program CLUSTAL W software [44]. The alignment was edited with the BioEdit software v7.0.0 and MEGA 3.1 [45,46]. Aligned sequences were analyzed in the Modeltest program to identify the best fit-model of nucleotide substitution for phylogenetic reconstruction; in all the analysis the Tamura and Nei (TrN+I+G) was the best model [47]. The best fit-model was selected under the hierarchical likelihood ratio test (hLTR). The phylogenetic relationships among strains were reconstructed by the neighbor-joining (NJ) and maximum parsimony (MP) methods using the PAUP 4.0B10 program [48].

### Structural analysis and comparisons

In order to identify location of the amino acid residues in the E protein the putative E protein structure of different isolates were compared with the E protein structure of DENV-3 deposited in the Protein Data Bank (PDB) under the access code 1UZG[32]. Analysis of the structures and construction of the illustrations were done using the graphical program Pymol [49].

## Competing interests

The authors declare that they have no competing interests.

## Authors' contributions

AAA, FTA, DJ, HLA, LCA, NAN, LTF and VHA conceived of the study, and participated in its design and coordination. All authors read and approved the final manuscript.

## Supplementary Material

Additional file 1 **Database of the E protein gene sequences analyzed in this study**. The file provides details on all the sequences including in this study. Click here for file

Additional file 2 **Alignment of nucleotide and amino acid sequences of the E protein of the 361 strains of DENV-3**. The file provides details on all the variable sites distributed in the E protein gene. Click here for file

Additional file 3 **Nucleotide and amino acid substitutions in the 95 informative sites of the E gene of DENV-3**. The file provides details on nucleotide and amino acid substitutions in the informative sites of the E gene of DENV-3. Click here for file

Additional file 4 **A stereoscopic drawing of the tertiary structure of E protein indicating the location of the amino acid residues**. Domains I, II and III are colored in red, yellow and blue, respectively. The overlapping amino acids are in gray. A) Location of amino acids that characterize the genotypes. B) Location of amino acids that characterize the lineage I and II of the genotype I. C) Location of amino acids that characterize the groups within the lineage I of genotype II. D) Location of amino acids that characterize the groups within the lineage I of genotype III. Click here for file

Additional file 5 **Comparison of the E protein amino acid sequence of the 361 viruses**. Details on the frequency of amino acids. Click here for file

Additional file 6 **Comparison of E the protein amino acid sequence of the Cuba strains and Puerto Rico**. Sequence of isolates from Cuba and Puerto Rico, which showed differences of amino acids in several sites of the E protein. Click here for file
