# Supplementary material for: Genetic diversity of the E Protein of Dengue Type 3 Virus
Source: Virol J. 2009 Jul 23;6:113. doi: 10.1186/1743-422X-6-113 (PMC2720943; doi:10.1186/1743-422X-6-113)
Supplement: Additional file 4 — A stereoscopic drawing of the tertiary structure of E protein indicating the location of the amino acid residues. Domains I, II and III are colored in red, yellow and blue, respectively. The overlapping amino acids are in gray. A) Location of amino acids that characterize the genotypes. B) Location of amino acids that characterize the lineage I and II of the genotype I. C) Location of amino acids that characterize the groups within the lineage I of genotype II. D) Location of amino acids that characterize the groups within the lineage I of genotype III. [file 1743-422X-6-113-S4.ppt]

## Slide 1
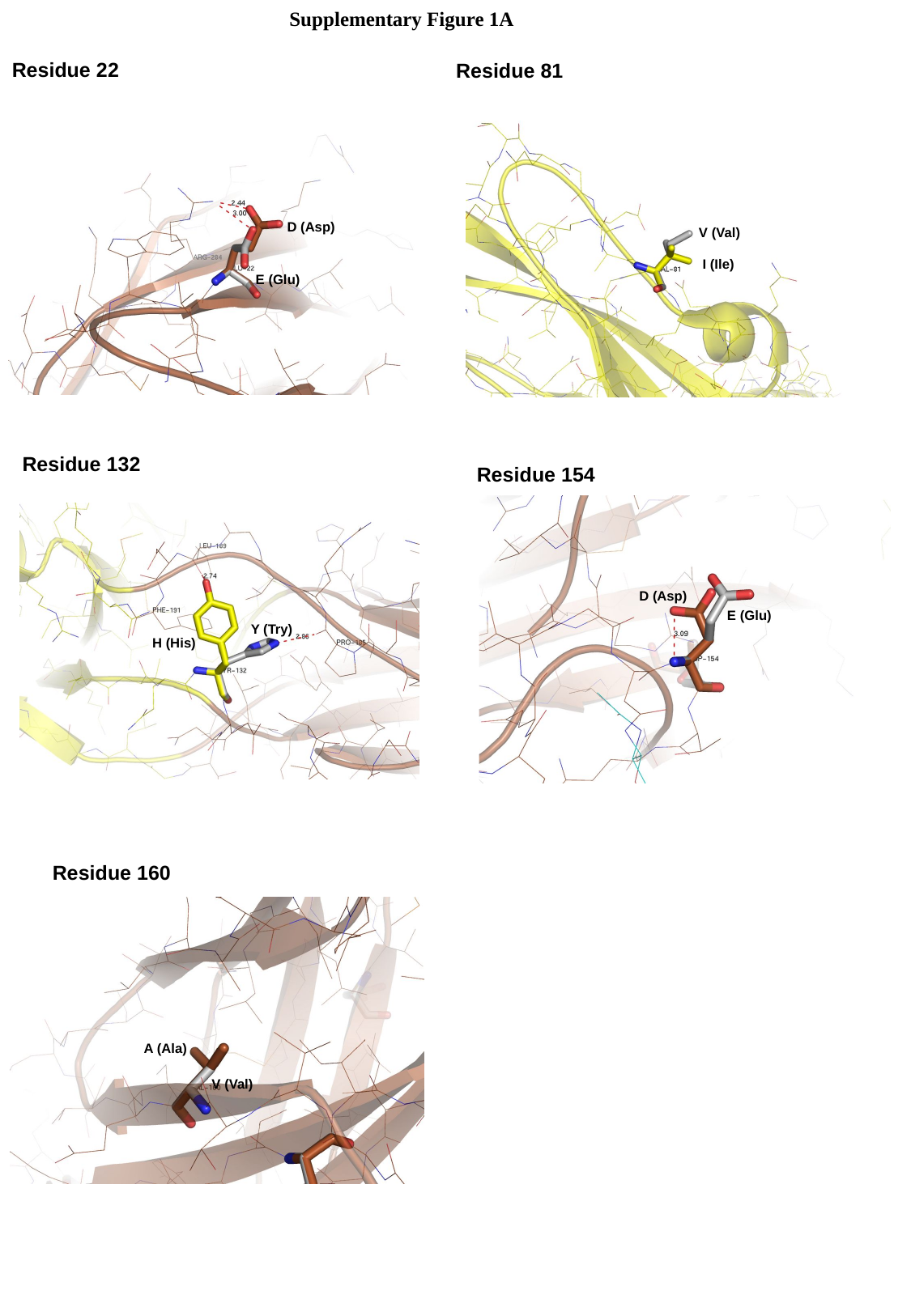

Supplementary Figure 1A
Residue 22
D (Asp)
E (Glu)
Residue 81
V (Val)
I (Ile)
Residue 132
Y (Try)
H (His)
Residue 154
D (Asp)
E (Glu)
Residue 160
A (Ala)
V (Val)

## Slide 2
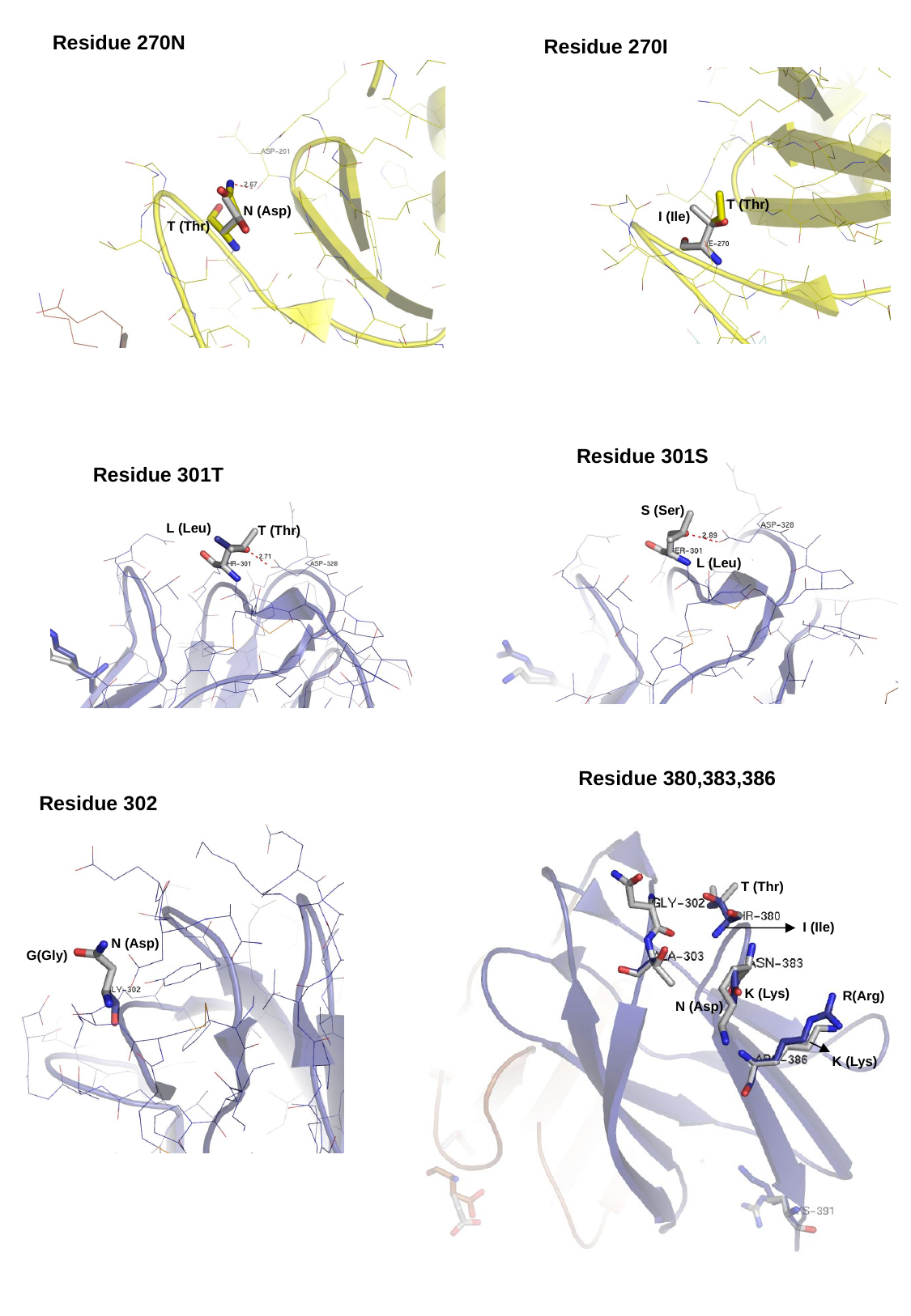

Residue 270N
N (Asp)
T (Thr)
Residue 270I
T (Thr)
I (Ile)
Residue 301S
S (Ser)
L (Leu)
Residue 301T
L (Leu)
T (Thr)
Residue 380,383,386
T (Thr)
I (Ile)
K (Lys)
R(Arg)
N (Asp)
K (Lys)
Residue 302
N (Asp)
G(Gly)

## Slide 3
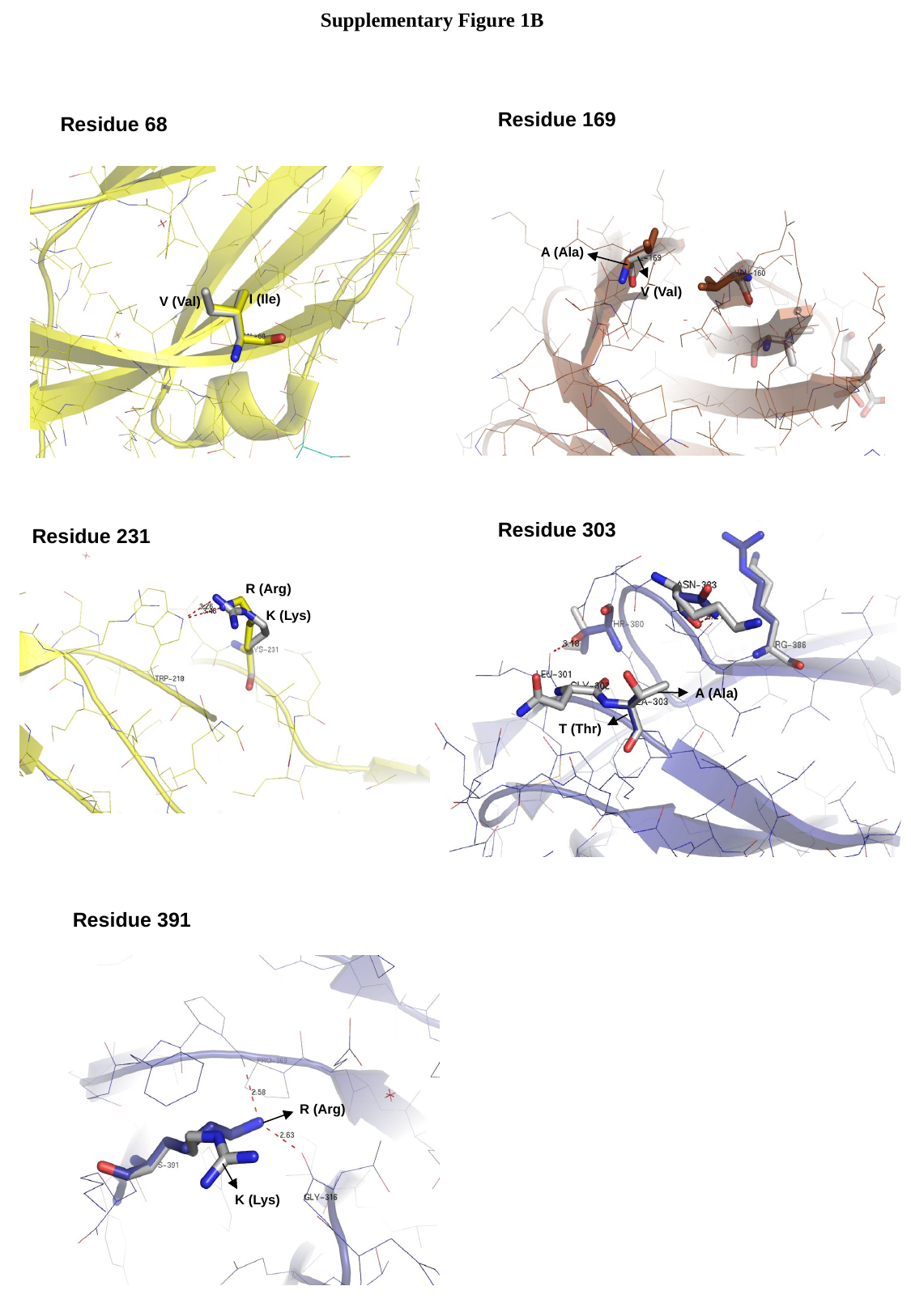

Supplementary Figure 1B
Residue 169
A (Ala)
V (Val)
Residue 68
I (Ile)
V (Val)
Residue 231
R (Arg)
K (Lys)
Residue 303
A (Ala)
T (Thr)
Residue 391
R (Arg)
K (Lys)

## Slide 4
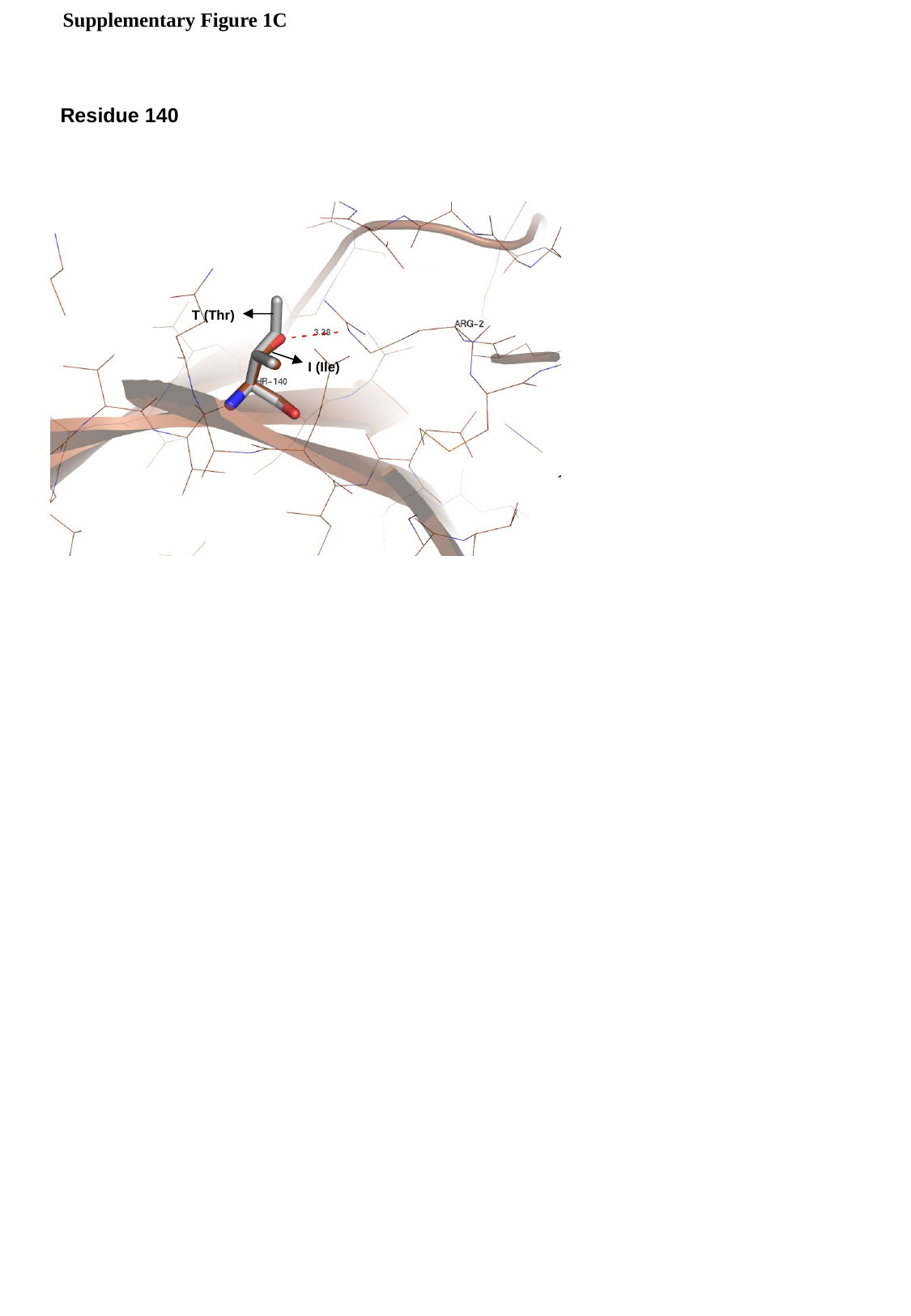

Supplementary Figure 1C
Residue 140
T (Thr)
I (Ile)
